# Prognostic Significance of Lymphoid Enhancer-Binding Factor-1 Expression in Egyptian Adult B-Acute Lymphocytic Leukemia Patients

**DOI:** 10.4274/tjh.2013.0140

**Published:** 2015-02-15

**Authors:** Rabab M. Aly, Ansaf B. Yousef

**Affiliations:** 1 Mansoura University Faculty of Medicine, Department of Clinical Pathology, Mansoura, Egypt; 2 Mansoura University Faculty of Medicine, Department of Internal Medicine, Mansoura, Egypt

**Keywords:** Lymphoid enhancer-binding factor-1, Acute lymphoblastic leukemia, prognosis, Wnt

## Abstract

**Objective:**

Lymphoid enhancer-binding factor-1 (LEF-1) is a key transcription factor of wingless-type (Wnt) signaling in various tumors and it is associated with a number of malignant diseases such as leukemia. We explored the expression profile of LEF-1 in acute lymphoblastic leukemia (ALL) and determined its specific prognostic significance in this disease.

**Materials and Methods:**

We studied LEF-1 expression in 56 newly diagnosed B-acute ALL adult patients using real-time quantitative polymerase chain reaction to investigate whether LEF-1 expression was associated with clinical patient characteristics and treatment outcomes.

**Results:**

High LEF-1 expression was associated with significantly poorer disease-free survival (p=0.03) and overall survival (p=0.005). Patients with high LEF-1 expression had a significantly higher relapse rate compared with low LEF-1 expression (p=0.01).

**Conclusion:**

We provide evidence that high LEF-1 expression is a prognostic marker in adult B-acute ALL patients.

## Abstract (tr)

**Amaç:**

Lenfoid enhansır-bağlayıcı faktör-1 (LEF-1), çeşitli tümörlerde wingless-type (Wnt)’yi işaret eden ana transkripsiyon faktörü olmakla birlikte lösemi gibi birçok ölümcül hastalıkla da ilişkilendilir. Biz bu çalışmada, LEF-1’in akut lenfoblastik lösemideki (ALL) ekspresyon profilini araştırdık ve LEF-1’in bu hastalık genelindeki özel prognostik önemini saptadık.

**Gereç ve Yöntemler:**

B-ALL hastalığı tanısı yeni konmuş 56 yetişkin hastada LEF-1 ekspresyonunu çalıştık ve LEF-1 ekspresyonunun klinik hasta özellikleriyle ve tedavi sonuçlarıyla ilişkili olup olmadığını araştırmak için gerçek-zamanlı kantitatif polimeraz zincir reaksiyonu uyguladık.

**Bulgular:**

Yüksek LEF-1 ekspresyonu olan hastalarda hastalıksız sağkalım (p=0,03) ve genel sağkalım (p=0,005) daha kısaydı. Düşük LEF-1 ekspresyonlu hastalarla kıyaslandığında, yüksek LEF-1 ekspresyonunlu hastalarda anlamlı olarak daha yüksek nüks oranları gözlendi (p=0,01).

**Sonuç:**

Bu çalışma sonucunda, yüksek LEF-1 ekspresyonunun yetişkin B-ALL hastalarında prognostik belirteç olduğuna yönelik kanıt sunuyoruz.

## INTRODUCTION

The outcome of adult B-precursor acute lymphoblastic leukemia (B-ALL) has considerably improved because of identification of clinical and genetic risk factors stratifying patients into different treatment groups [1]. Common risk factors in B-ALL include white blood cell (WBC) count, the immunophenotype of B-ALL, response to induction therapy, level of minimal residual disease, age, and cytogenetic as well as molecular genetic aberrations [2]. High-risk cytogenetics in B-ALL mainly comprise the Philadelphia chromosome with balanced translocation t(9; 22) (q34; q11) and the BCR-ABL fusion gene in approximately 20% to 36% of cases [3] and translocation t(4; 11) (q21; q23) with the MLL-AF4 fusion gene in approximately 6% to 9% of cases [4]. Patients lacking clinical and molecular risk factors are considered to be of standard risk. The relapse rate is still approximately 40% to 50% and relapse is not predictable with pretreatment markers in the standard-risk patient group without established risk factors [5,6].

The identification of new prognostic factors is of particular interest for this subgroup of ALL. Moreover, it may help to develop novel targeted therapies for these patients. It was previously demonstrated that ordered expression of lymphoid enhancer-binding factor-1 (LEF-1) is necessary for normal hematopoietic stem cell function in mice, and that LEF-1 overexpression induces acute myeloid leukemia (AML) [7]. LEF-1 plays a crucial role in the development of B and T lymphocytes as well as neutrophilic granulocytes [8,9]. In different hematologic malignancies, including lymphomas, chronic lymphocytic leukemia, and ALL and AML [10,11,12,13,14], LEF-1 was found to be highly expressed. In vitro studies revealed a prosurvival effect of LEF-1 in an AML1-ETO-positive leukemic cell line, primary chronic lymphocytic leukemia cells, and murine T-cell lymphomas [11,12,15].

LEF-1 is a member of the lymphoid enhancer factor/T-cell factor (LEF/TCF) family (LEF-1, TCF-1, TCF-3, and TCF-4) of the HMG transcription factors [16]. LEF-1 acts as a central transcription mediator of Wingless-type (Wnt) signaling, regulating cell cycle, and growth-relevant genes like cyclin D1 and c-myc [17,18,19]. The Wnt pathway has been implicated in leukemic transformation and was shown to promote proliferation and survival of leukemic cells in vitro [15,20,21]. Studies on LEF-1 in hematopoietic development have been mainly restricted to the lymphoid lineages, where LEF-1 has functions in T-cell development and affects proliferation and apoptosis in pro-B cells [8]. Recent reports, however, also discuss specific functions of LEF-1 independent of Wnt signaling, suggesting a more complex role of LEF-1 in the development of hematopoietic tissues [15].

The human LEF-1 gene spans at least 140 kb and contains 12 exons and 11 introns, with a large third intron (about 75 kb) that may contain an alternative exon. The LEF-1 gene encodes at least 2 isoforms. LEF-1 is a sequence-specific DNA binding protein that binds to a functionally important site in the T-cell receptor-alpha enhancer through an HMG domain and confers maximal enhancer activity [22].

## MATERIALS AND METHODS

### Patients

Fifty-six Egyptian patients (43 males and 13 females) diagnosed with B-ALL at the Oncology Center of Mansoura University Hospital were enrolled in this study. The study was approved by the Institutional Review Board of the Mansoura University Hospital. All patients gave informed consent to morphological and molecular examination. Diagnosis of ALL was based on morphologic and immunophenotypic criteria. Heparinized bone marrow (BM) and peripheral blood samples were collected prior to treatment. Minimal residual disease was effectively monitored by use of BM samples. Clinical and hematological parameters were determined. All included patients received the same treatment protocol, approved by the oncology team of the oncology center. It included 6 weeks of induction, 2 weeks of consolidation, and 120 weeks of continuation therapy. Induction therapy consisted of vincristine (VCR), daunomycin, asparaginase, etoposide (VP-16), and aracytin (Ara-C) in addition to triple intrathecal (IT) therapy including 2 courses of high-dose methotrexate (HDMTX), 6-mercaptopurine (6-MP), and dexamethasone (Dex). Continuation therapy consisted of extended triple IT therapy and 15 cycles of an 8-week course of VP-16+cyclophosphamide (CTX), 6-MP+MTX, MTX+Ara-C, Dex+VCR, VP-16+Ara-C, 6-MP+HDMTX, VP-16+Ara-C, and Dex+VCR. During continuation therapy, reinduction was given in the form of VCR, daunomycin, Dex, HDMTX, 6-MP, and triple IT therapy.

Complete remission was defined as mononuclear BM containing less than 5% blast cells and showing evidence of normal maturation of other marrow elements after induction chemotherapy. Relapse was defined by the appearance of more than 5% lymphoblasts in a single BM aspirate or leukemic cell infiltration in extramedullary organs [23].

### Methods

#### RNA Extraction and cDNA Synthesis

High-quality RNA was extracted using the RNeasy Mini Kit in accordance with the manufacturer’s instructions (QIAGEN, Valencia, CA, USA). The concentration, quality, and purity of RNA were measured by UV spectrophotometer at 260/280 nm. The integrity and the size distribution of total RNA were checked by electrophoresis on 1.5% agarose gel. cDNA synthesis reaction was performed with 25 µL of total RNA, 2.5 µL of reverse transcriptase, 5 µL of RT, 4 µL of dNTPs, 5 µL of random primers, and 8.5 µL of water. This mixture was then incubated at 42 °C for 1 h. The reaction was inactivated by heating at 95 °C for 5 min.

#### Analyses of Gene Expression by Real-Time-Polymerase Chain Reaction

The LEF-1 gene and reference gene GAPDH were quantified according to the real-time quantitative polymerase chain reaction (RT-PCR) method using the Applied 7700 sequence detection system (TaqMan; PerkinElmer Applied Biosystems, Foster City, CA, USA). RT-PCR was performed in a MicroAmp optical 96-well plate with 10 µL of the cDNA solution, 1.0 µM of forward primer, 1.0 µM of reverse primer, 10 µL of dH2O, 0.5 µL of probe, and 25 µL of universal master mix.

The sequences of forward and reverse primers for measurement of LEF-1 expression were as follows: LEF-1 probe, 5-FAM-CCAGATTCTTGGCAGAAGGTGGCAT-TAMRA; LEF-1 forward, 5-AATGAGAGCGAATGTCGTTGC; and LEF-1 reverse, 5-GCTGTCTTTCTTTCCGTGCTA.

The sequence of the primers and probe of the GAPDH control were: GAPDH forward primer, 5’-GAAGGTGAAGGTCGGAGTC-3’; GAPDH reverse primer, 5’ GAAGATGGTGATGGGATTTC-3; and GAPDH probe, VIC-CAAGCTTCCCGTTCTCAGCC-TAMRA.

All samples were analyzed in duplicate. The variation of the duplicate measurements was extremely small compared to the variation between different samples. For each patient, the relative mRNA expression levels of LEF-1 were calculated using the comparative cycle time (Ct) method [24]. The target PCR Ct value, which is the cycle number at which emitted fluorescence exceeds 10 times the standard deviation of baseline emissions, was normalized to the GAPDH PCR Ct value by subtracting the GAPDH Ct value from the target PCR Ct value. The mRNA expression level relative to GAPDH for each target PCR was calculated using the following equation: relative mRNA expression= 2-(Ct target-Ct GAPDH).

## STATISTICAL ANALYSIS

SPSS 15.0 for Windows (SPSS Inc., Chicago, IL, USA) was used for all calculations. Clinical features across groups were compared using the χ2 or 2-sided Fisher exact test for categorical data and the nonparametric Mann-Whitney U test for continuous variables. Survival curves were calculated by the Kaplan-Meier method. Multivariate analyses were performed using the Cox proportional hazards model for survival, including the following variables in the full model: LEF-1 expression, age, WBC count, and immunophenotype. P<0.05 was considered statistically significant.

## RESULTS

We determined LEF-1 expression in 56 patients with newly diagnosed B-ALL. There were no significant correlations between LEF-1 expression levels and clinical, laboratory, or immunophenotypic characteristics (Table 1). Based on the detection of LEF-1 median expression level (1.73), patients were divided into the low-expression group (LEF-1 expression level of <1.73) and high-expression group (LEF-1 expression level of >1.73).

In the Cox regression analysis, LEF-1 expression was the most significant prognostic factor for disease-free survival (DFS) (p=0.001); the other significant factors predicting DFS were age and immunophenotype (Table 2).

We analyzed the influence of LEF-1 expression on the prognosis of B-ALL patients. Patients with high LEF-1 expression, as compared to patients with low LEF-1 expression, showed significantly lower remission rates (p=0.02). Patients with high LEF-1 expression were significantly associated with a higher relapse rate (p=0.01, Table 3).

Table 3 also showed that, at 3 years, the estimated overall survival (OS) was 58.9% in patients with low LEF-1 expression, which was higher than in patients with high expression (33.9%) (p=0.005). Adverse prognosis associated with high LEF-1 expression was observed in terms of 3-year DFS (high LEF-1, 23.4% vs. low LEF-1 expression, 51.0%; p=0.03).

## DISCUSSION

The prognostic markers in B-ALL have a significantly important role in the development of new molecular therapies for these patients. In the current study, we have used a quantitative real-time PCR assay to analyze the role of LEF-1 expression in patients with B-ALL.

In this study, we have identified high LEF-1 expression as an independent prognostic factor associated with a high risk of relapse and lower DFS in B-ALL patients. High LEF-1 expression was independently prognostic for lower OS, with a 3-year survival rate of only 33.9% for patients with high LEF-1 expression compared to 58.9% in the patient group with low LEF-1.

Several studies found effects of LEF-1 expression on the degree of malignancy in neoplastic diseases. LEF-1 appears to mediate tumor growth and invasion ability in androgen-independent prostate cancer [25]. Moreover, Nguyen et al. found that LEF-1 also mediates cell invasion in breast cancer [26]. Deregulated LEF-1 expression may also be an important step in the development of neoplastic diseases. LEF-1 is involved in B and T lymphocyte development [8,27]. The causes and effects of abnormal LEF-1 expression likely depend on the cellular context and differentiation stage. The diverse functions of LEF-1 in normal and malignant hematopoiesis are reflected by recent reports that high LEF-1 expression is associated with inferior outcomes in B-ALL [28]. Metzeler et al. reported a larger increase of the expression of LEF-1 in ALL samples than in AML, reflecting the higher expression of the transcription factor in lymphoid tissue [29]. Several studies found that deregulated expression of LEF-1 could induce B-ALL [7,8].

Our study showed no significant association of clinical characteristics and high LEF-1 expression. Conversely, in patients with T-ALL, LEF-1 was characterized by distinctive clinical features, including a younger age at diagnosis and a trend toward improved OS in children treated with contemporary T-ALL combination therapy [30].

Current risk stratification is based primarily on clinical variables, immunophenotyping, detection of cytogenetic or molecular lesions, and early response to therapy [31]. Importantly, our study shows that high LEF-1 expression was also associated with poor DFS. This was in agreement with Kühnl et al., who found that high LEF-1 expression identifies B-ALL patients with inferior DFS [28].

In multivariate analysis, the association between LEF-1 expression and outcome showed that high LEF-1 expression is more significant than other prognostic factors for DFS, such as for age, leukocyte count at presentation, or immunophenotype; this indicates that high LEF-1 expression at diagnosis might be useful in identifying patients with a high risk of treatment failure.

A study of T-ALL patients with inactivating LEF-1 mutations showed a trend toward a favorable OS [30]. Conversely, in patients with myelodysplastic syndrome, advanced disease and poor prognosis were associated with downregulation of LEF-1, probably reflecting the impaired maturation of myeloid progenitors associated with loss of LEF-1 function [15,32]. In leukemic cells, LEF-1 enhanced self-renewal properties and survival in vitro and was shown to confer leukemogenic potential in a mouse model [7,12,15]. Furthermore, patients with high LEF-1 expression should be considered for new molecular directed therapies, especially agents targeting the Wnt pathway [33].

In summary, we provide evidence that high LEF-1 expression has adverse prognostic significance and thus may provide a valuable new approach to molecular-targeted therapy in B-ALL patients.

## Figures and Tables

**Table 1:**
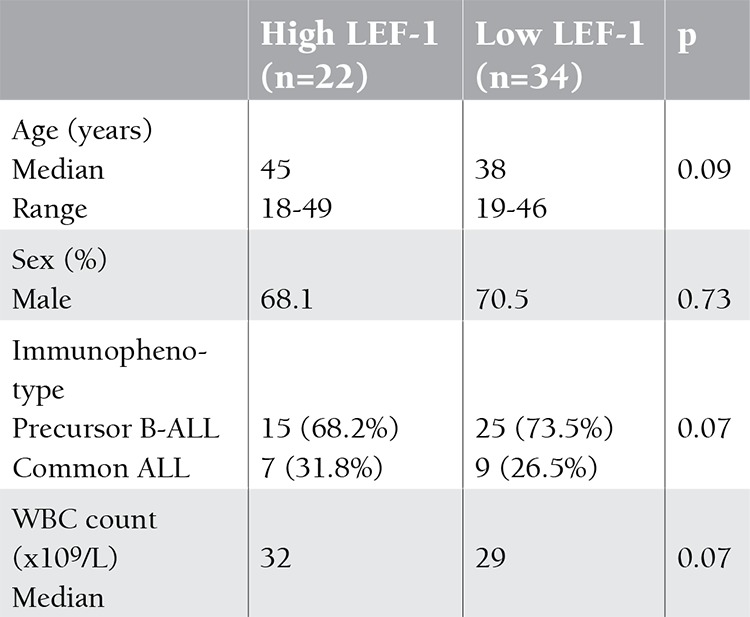
LEF-1 expression and clinical characteristics in acute lymphoblastic leukemia (ALL) cases.

**Table 2:**
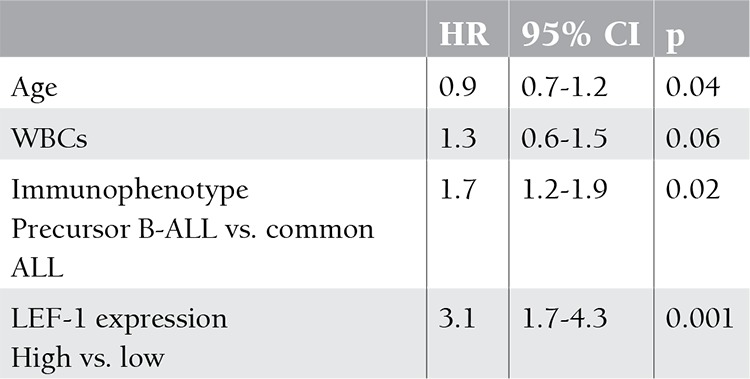
Multivariate analysis of LEF-1 expression for disease-free survival (DFS) in acute lymphoblastic leukemia (ALL) patients.

**Table 3:**
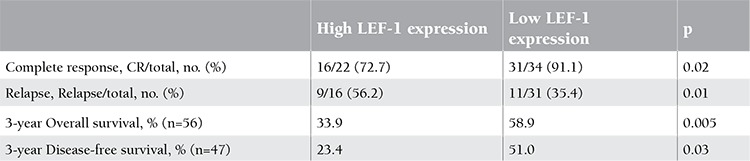
LEF-1 expression and clinical outcome.
